# Robust tribo-mechanical and hot corrosion resistance of ultra-refractory Ta-Hf-C ternary alloy films

**DOI:** 10.1038/s41598-017-03181-2

**Published:** 2017-06-08

**Authors:** Luis Yate, L. Emerson Coy, Willian Aperador

**Affiliations:** 1 CIC biomaGUNE, Paseo Miramón 182, 20009 Donostia-San Sebastian, Spain; 2 NanoBioMedical Center, Adam Mickiewicz University, Umultowska 85, 61-614 Poznan, Poland; 3 School of Engineering, Universidad Militar Nueva Granada, Carrera 11 #101-80, 49300 Bogotá, Colombia

## Abstract

In this work we report the hot corrosion properties of binary and ternary films of the Ta-Hf-C system in V_2_O_5_-Na_2_SO_4_ (50%wt.-50%wt.) molten salts at 700 °C deposited on AISI D3 steel substrates. Additionally, the mechanical and nanowear properties of the films were studied. The results show that the ternary alloys consist of solid solutions of the TaC and HfC binary carbides. The ternary alloy films have higher hardness and elastic recoveries, reaching 26.2 GPa and 87%, respectively, and lower nanowear when compared to the binary films. The corrosion rates of the ternary alloys have a superior behavior compared to the binary films, with corrosion rates as low as 0.058 μm/year. The combination and tunability of high hardness, elastic recovery, low nanowear and an excellent resistance to high temperature corrosion demonstrates the potential of the ternary Ta-Hf-C alloy films for applications in extreme conditions.

## Introduction

Thermal barrier or ultra-refractory materials have been widely used as protective coatings for components exposed to high temperatures (from 550 to 900 °C) in aerospace and applications such as turbine blades and steam generators in power plants^1–4^. Generally, the most used materials used for thermal barrier coatings are metal superalloys^1, 5^ and yttria-stabilized-zirconia (YSZ) and YSZ-based systems^2, 6, 7^. Those materials have excellent chemical stability at high temperatures, good anti-corrosion properties and low thermal conductivity^7^, however, most of the superalloys and YSZ-based materials are brittle and lack of high hardness and wear resistance which restrict the applications where the materials are subject to high stresses and loads.

Transition metal carbides have received a great attention in the last years due to the combination and tunability of their chemical and physical properties; high hardness, low wear, high electrical conductivity, high melting points, good chemical stability and corrosion resistance. Among binary metal carbides, tantalum carbide (TaC) and hafnium carbide (HfC), also known as ultra-refractory carbides^8^, are of particular interests due to the extremely high melting points, near 4000 K^9, 10^ and relatively high hardness over 20 GPa^11, 12^. It has been recently reported that the ternary Ta-Hf-C alloy, formed by a solid solution of TaC and HfC binary carbides, presents the highest melting point temperature for any solid, at around 4215 K^10^. In addition, it has been reported that compared with transition metal binary materials, the corresponding ternary alloys were found to exhibit better mechanical properties^13^. Due to their attractive properties, binary and ternary alloys from the Ta-Hf-C system are promising materials for applications in extreme conditions under high temperatures and loads and corrosive environments. Pan *et al*.^8^ have reported the outstanding thermodynamic behavior of the Ta_x_-Hf_(x-1)_-C system in the entire range of compositions, x = [0, 1], and temperature, T = [500, 4100] Celsius, but there are no studies describing the hot corrosion behavior of the Ta-Hf-C ternary alloys.

The objective of this research was to measure the tribo-mechanical and hot corrosion properties on binary and ternary Ta-Hf-C thin films deposited by non-reactive magnetron sputtering. The hot corrosion properties were studied by potentiodynamic curves in presence of a mixture of pentoxide vanadium, V_2_O_5_, and sodium sulfate, Na_2_SO_4_, analytic grade to concentrations of 50:50 (in weight) in a high temperature furnace coupled with an electrochemical cell. The nature of the corrosion process was studied by using scanning electron microscopy (SEM), energy dispersive X-ray spectrometry (EDS) and X-ray diffraction and showed the protective potential of the Ta-Hf-C films.

## Methods

### Materials preparation

Ta-Hf-C alloy films were grown on silicon (100) and AISI D3 steel substrates by non reactive magnetron sputtering using an AJA-ATC 1800 system with a base pressure of 10^−7^ Pa. The P-type silicon (100) substrates with resistivity around 1–10 Ohm·cm and thickness of 380 μm were acquired from University Wafer-USA. The AISI D3 steel was cut in disks of 12.7 mm in diameter and 4.8 mm in thickness and with a mirror-polished finish. The deposition of the films was done with three separate 5.08 cm elemental targets, with a purity of 99.999% for carbon (graphite) and 99.95% for both Ta and Hf targets, in a confocal configuration at a pressure of 0.4 Pa of pure Ar. All targets were acquired from Demaco-Holland. The samples were grown with a negative bias voltage of 50 V with the substrate holder at 300 °C and rotating at 80 RPM during a deposition time of 1 hour. The distance between target and substrates was about 15 cm. Prior to deposition, the substrates were sputter cleaned with a negative bias of 190 V (25 W) in an Ar atmosphere (4 Pa) for 10 min. In order to improve the adhesion of the films to the substrates, a Ta-Hf metallic layer of ∼20 nm was deposited on the substrates at a radio frequency (r.f.) power of 100 W for each target at a substrate holder temperature of 300 °C without bias voltage. Ta-Hf-C films with different compositions and thicknesses between 0.2 and 0.3 μm were obtained by varying the Ta and Hf target (r.f.) power (100–0, 70–30, 30–70 and 0–100 W, and hereafter referred to as TaC, 70TaC-30HfC, 30TaC-70HfC and HfC, respectively) while keeping the carbon target direct current (d.c.) power constant at 380 W.

### Chemical and structural characterization

Film thicknesses were determined by SEM with a JEOL JSM-6490LV microscope. The topography of the films was studied by atomic force microscopy (AFM, Nanoscope V Multimode atomic force microscope, Bruker) on an area of 500 nm × 500 nm. The error in the film thickness and roughness was obtained as the standard deviation of at least three different measurements.

X-ray photoelectron spectroscopy (XPS) analyses were performed by means of a SAGE HR100 (SPECS) with a non monochromatic source (Mg Kα 1283.6 eV) after a short etching of the sample surface with Ar^+^ ions at 3 kV energy in order to remove contamination. The spectra resolution was around 1.0 eV, previously measured using the full width at half maximum (FWHM) of the 3d_5/2_ line of Ag. All measurements were made in ultra high vacuum (UHV) at a chamber pressure of around 8·10^−8^ mbar.

X-ray diffraction (XRD) analysis was conducted for structural investigations of the films using an Empyrean PANalytical X-ray diffractometer with Cu Kα radiation (λ = 1.54060 Å) in the Bragg-Brentano configuration at a counting time of 1 second per step and 0.03° step size. The identification of the phases was performed with the X’Pert High Score software through the ICCD database.

### Mechanical and tribological characterization

The nanohardness of the films was measured by a tribo-indenter (Hysitron TI 950 TriboIndenter) using a Berkovich diamond indenter at a maximum load of 7500 μN, indentation times were set as: load (5 s), load holding (2 s) and unload (5 s). Hardness values were determined from the load-displacement curves by the Oliver–Pharr method^14^ and values extracted at different penetration depth were fitted to the Korsunsky equation^15^ in order eliminate substrate contribution. The elastic recovery of the samples was determined as the percentage of the residual imprint compared to the total displacement of the load-displacement curves. Nanowear tests were also performed with the tribo-indenter over areas of 1 µm × 1 µm using a Berkovich tip at a constant load of 0.1 mN and a frequency of 2 Hz (20 µm/s). The displaced volume was calculated from the height variation between the removed sections and pristine areas using Gwyddion software^16^.

### Hot corrosion characterization

**Figure 1:**
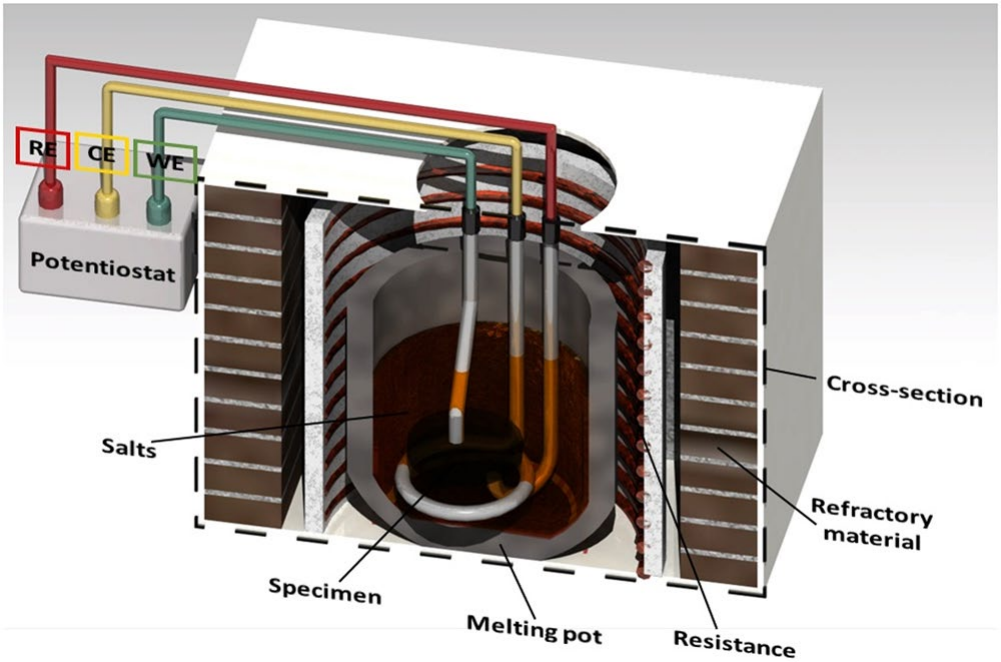
Schematic representation of the high temperature electrochemical cell used for the measurements. RE, CE and WE are the reference, counter and working electrodes, respectively.

The electrochemical tests were performed on a high temperature furnace coupled with an electrochemical cell with the films deposited on the steel substrates, Fig. 1. The electrochemical cell consists of an array of 3 electrodes: the working, reference and auxiliary (counter) electrode. The working electrode was welded to a platinum wire which served as an electrical conductor, this wire was also placed inside a ceramic tube of mullite industrial grade so only the specimen was exposed to the corrosive environment. The reference and counter electrodes were built with high purity platinum wire, and covered with a mullite tube resistant to high temperatures. The hollow parts between the specimen and the ceramic tube were sealed with refractory cement as well as the spots of the other electrodes.

Corrosion tests were performed using a mixture of pentoxide vanadium, V_2_O_5_, (acquired from Alfa Aesar) and sodium sulfate, Na_2_SO_4_, (acquired from Merck Millipore) analytic grade to concentrations of 50:50 (in weight), which are common salt contaminants involved in hot corrosion and hot boilers^17^ and found in low-quality fuels at high temperatures^6^. The mixture was macerated during 45 minutes in agate mortar for homogenization, and then 30 g were placed in a porcelain crucible that subsequently was inserted inside of the vertical furnace. The electrochemical cell was placed in the furnace, slowly increasing the temperature from room temperature to 700 °C, temperature at which the electrochemical tests were performed. The setup of the high temperature cell used for the electrochemical analysis can be seen in Fig. 1. The internal temperature was monitored by a Type K thermocouple connected to a PID, temperature controller. To resemble a boiler environment a mixture of 99%O_2_-1%SO_2_ was used as oxidizing gas.

Polarization curves were obtained by applying a potential of ±200 mV at a scan speed of 1 mV/s in potentiodynamic form. Corrosion potential and rate were obtained through Tafel extrapolation using a Gamry Instruments potentiostat. The potential corrosion values (E_corr_) and the corrosion current density (I_corr_) were determined through the extrapolation Tafel method using the software Gamry Echem Analyst. The anodic and cathodic polarization curves were obtained after 3 hours of exposure to the corrosive medium, time at which the corrosion potential stabilized. All electrochemical experiments were repeated at least once and the errors in the obtained parameters were calculated as the standard deviation of the different measurements.

Finally, the surface of the samples after the electrochemical test was studied by scanning electron microscopy (SEM) and energy dispersive X-ray spectroscopy (EDS) using a Zeiss EVO MA10 SEM microscopy coupled with an electron analyzer and grazing incidence X-ray diffraction (GI-XRD) at a grazing angle of 2° using an Empyrean PRO PANalytical X-ray diffractometer with Cu Kα radiation (λ = 1.54060 Å) at a counting time of 2 second per step and 0.02° step size.

## Results and Discussion

### Microstructure, morphology and chemical composition

The thickness of the samples was determined by cross-section SEM and the values are listed in Table 1. It can be seen that the thickness increases as we go from the pure TaC to the pure HfC sample. The higher thickness in the samples as the r.f. power to the Hf target is increased can be attributed to the higher sputtering yield of the Hf (0.37) compared to the one of Ta (0.26) under similar Argon bombardment conditions^18^ (around 300 V of potential at a r.f. power applied to the target of 100 W).

**Table 1:** Thickness, RMS roughness obtained from AFM measurements and chemical compositions obtained from XPS.

Sample	Thickness (nm)	RMS roughness (nm)	Ta (at.%)	Hf (at.%)	C (at.%)	O (at.%)
TaC	200 ± 20	2.94 ± 0.35	58.8	0.0	33.4	7.8
70TaC-30HfC	285 ± 4	1.32 ± 0.06	41.6	14.1	37.8	6.5
30TaC-70HfC	295 ± 3	0.55 ± 0.04	12.2	46.1	35.1	6.5
HfC	300 ± 12	2.97 ± 0.29	0.0	63.0	29.1	8.0

**Figure 2:**
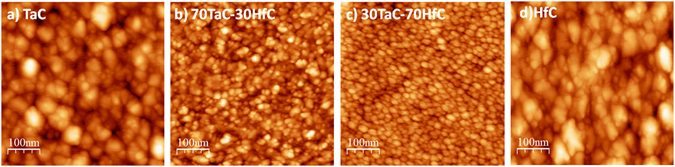
AFM images of the different Ta-Hf-C alloy films; (**a**) TaC, (**b**) 70TaC-30HfC, (**c**) 30TaC-70HfC and (**d**) HfC.

Figure 2 shows the AFM images with the details of the nano topography for the different Ta-Hf-C alloy films. The root mean squared (RMS) roughnesses of the samples, shown in Table 1, are below 2.97 nm. The lowest roughness value was found for the 30TaC-70HfC sample, Fig. 2c, which also shows homogeneous and well distributed grains, with grain sizes around 16 nm. In general, the ternary Ta-Hf-C alloys showed lower roughness and grain sizes than the binary films. This can be explained by the enhanced ion bombardment generated during the simultaneous sputtering of the Ta and Hf targets with different sputtering yields^19^ and the intrinsic nature of the binary and ternary carbides, in which the grain size is strongly influenced by the addition of extra elements, especially those who have chemical affinity or are known to alloy^20^.

The chemical compositions of the samples were obtained through XPS and are listed in the Table 1. It can be seen how the Ta and Hf contents vary as the r.f. power to the targets is varied. The metal content in the HfC film (63.0 at.%) is slightly higher than in the TaC (58.8 at.%) and is related to the higher sputtering yield for Hf. The oxygen content was below 8.0 at.% for all samples and its content can be attributed to the high affinity of metals for oxygen.

**Figure 3:**
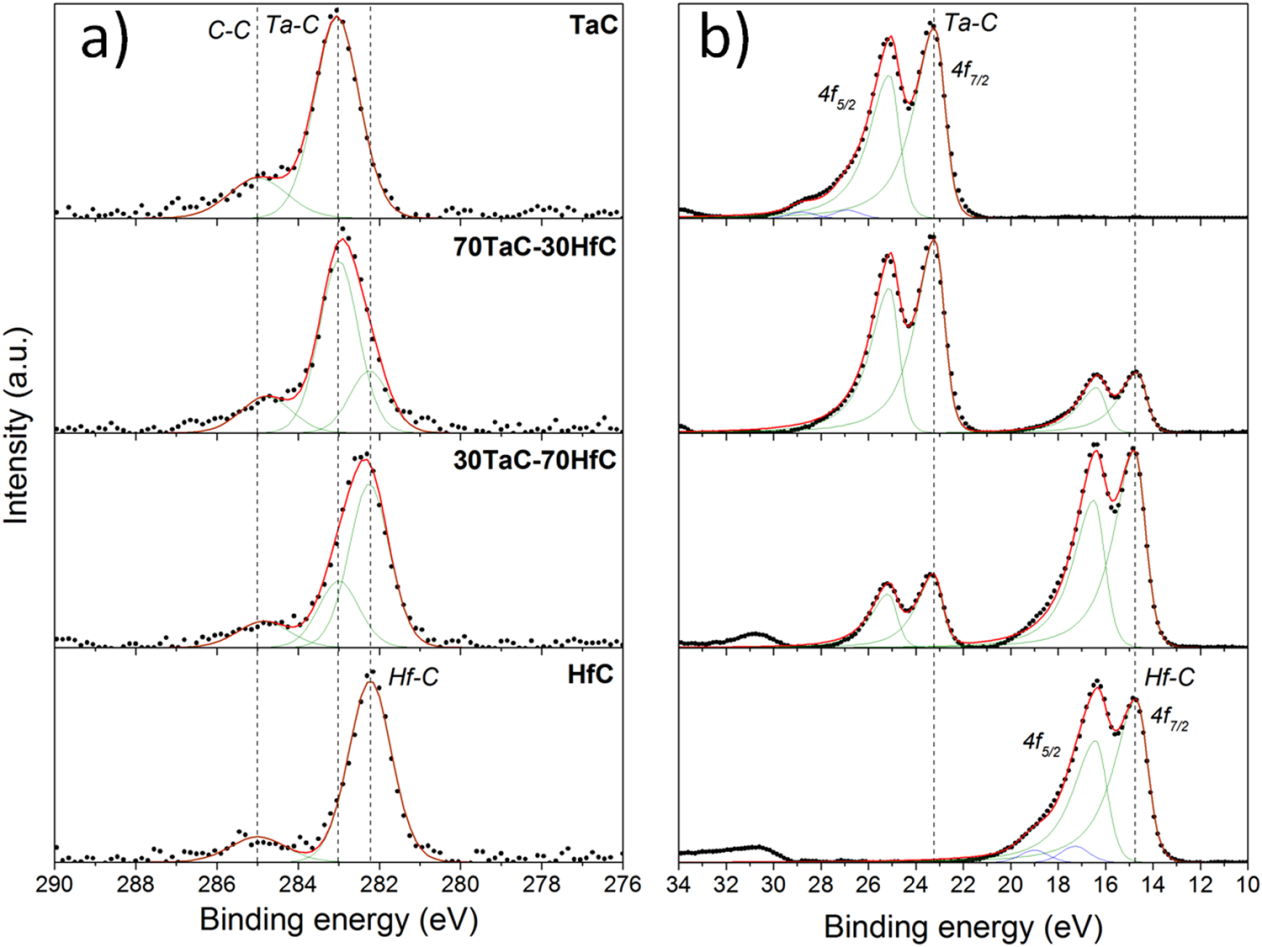
High resolution (**a**) C 1s and (**b**) Ta and Hf 4 f spectra for the different Ta-Hf-C alloy films showing the evolution of the samples as they change from pure TaC to HfC.

The high resolution C 1s and Ta 4f and Hf 4f spectra are shown in Fig. 3 and reveal the evolution in the bonding as the material change from pure TaC to HfC. The fitting of the C 1s spectra in Fig. 3a showed the presence of Ta-C bonds in the TaC binary film at a binding energy around 283.0 eV^21^, Hf-C bonds in the HfC sample at around 282.2 eV^22^, and the presence of both Ta-C and Hf-C bonds in the ternary alloy films. A minor amount of C-C bonds at around 285.0 eV^23^ was also detected. The Ta 4f and Hf 4f spectra in Fig. 3b also revealed the presence of Ta-C and Hf-C bonds in the samples. Due to the conductive characteristic of the samples where a distribution of conductive electrons are available for shake-up like events, the Ta 4f and Hf 4f spectra present asymmetry^24^ and were fitted with asymmetric shapes. The Ta 4f_7/2_ peak position in the TaC binary film was located at around 23.2 eV and correspond to Ta-C bonds. The Hf 4f_7/2_ peak in the HfC film was located at around 14.8 eV and it is attributed to Hf-C bonds^25^. The ternary alloys also showed the presence of both Ta-C and Hf-C with different proportions according to the different contents of Ta or Hf. Unlike the ternary alloy films, both binary samples showed the presence of very small peaks attributed to Ta-O and Hf-O bonds, with the Ta and Hf 4f_7/2_ peaks at around 26.9^26^ and 17.3 eV^27^, respectively. The absence of oxides and the lower amount of oxygen in the ternary films, compared to the binary ones, suggest the higher stability of the ternary carbide and its lower reactivity to oxygen.

**Figure 4:**
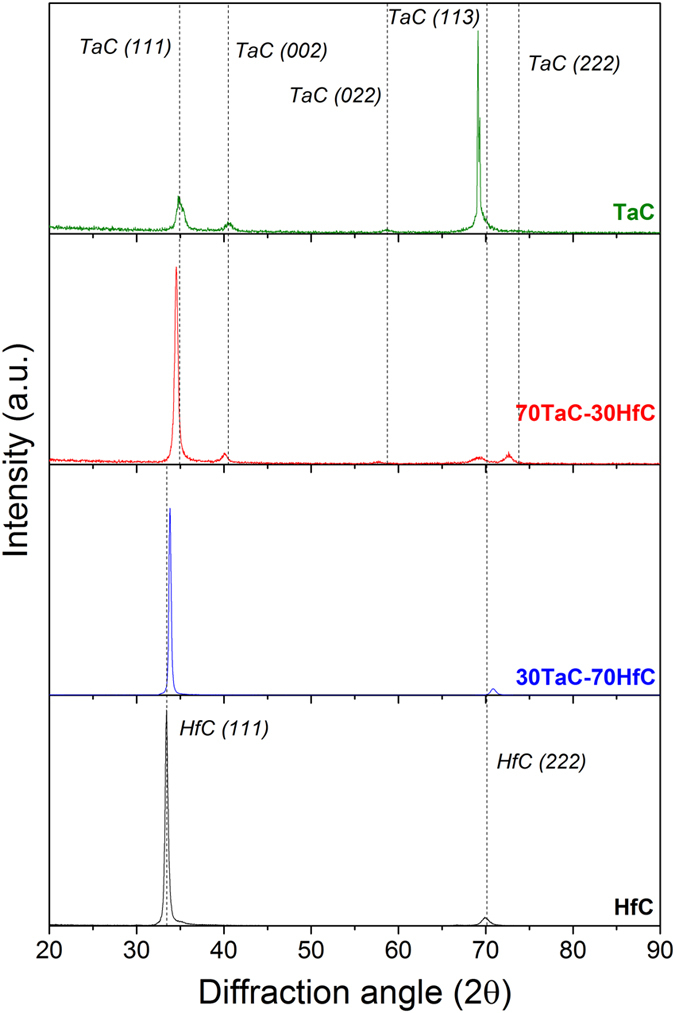
XRD patterns for the Ta-Hf-C deposited alloy films.

The crystalline structure of the samples was studied by X-ray diffraction. Figure 4 shows the XRD patterns of the deposited binary and ternary films. The patterns for the TaC and HfC binary films show the peaks associated to the cubic Fm3-m TaC and HfC phases, JCPDS-ICDD 00-035-0801 and JCPDS-ICDD 03-065-7113, respectively. The occurrence of the cubic TaC and HfC phases in the binary films with a good crystallinity and without secondary phases contrast to previously reported studies where Ta_2_C and TaC^28^ and metallic Hf and HfC^22^ mixed phases were obtained. In the case of the ternary alloys, the 70TaC-30HfC sample retains the structure of the TaC, only showing a change in the intensity of the diffracted peaks. Analogously, the 30TaC-70HfC sample also retains the structure of the HfC binary sample. As both the TaC and HfC materials present the same cubic Fm3-m structure and similar lattice parameter, 4.4470 and 4.6430 Å respectively, it is suggested that some Hf atoms are replacing some of the 4b Wyckoff sites of the Ta in the 70TaC-30HfC structure, and vice-versa for the 30TaC-70HfC sample. However, it is clear that both ternary samples show a very intense peaks from the (111) family, the position of which is placed half way between the theoretical HfC(111) and TaC(111), and can be indexed by the solid solution of Ta-Hf-C (JCPDS-ICDD 00-064-0146). This shows that in both ternary samples the Ta-Hf-C solid solutions have been formed, similarly to previous studies where the solid solution was obtained by plasma spray^9^.

### Mechanical and tribological properties

The hardness of the binary and ternary films is listed in Table 2. The hardness for the TaC and HfC are 12.3 and 18.5 GPa, respectively. These values fall into the range of hardness for TaC films deposited by magnetron sputtering^29^ and chemical vapor deposition^11^ with values between 9 and 22 GPa, and for HfC films deposited by pulsed laser ablation^12^ with hardness values around 20 GPa. On the other hand, the ternary alloys showed higher hardness values than the binary films, around 24.6 GPa for the 70TaC-30HfC and 26.2 GPa for the 30TaC-70HfC. Those values are also higher than the hardness reported for other authors for the binary TaC^11, 29^ and HfC^12^ films and more than two times higher than for Ta-Hf-C films deposited by the plasma spray technique, with reported values of around 12 GPa^9^. The higher hardness of the ternary alloys can be attributed to a combination of beneficial effects, i.e., the formation of a solid solution of the binary TaC and HfC carbides and to the lower grain sizes. In general, it is accepted that lower grain sizes lead to higher hardness according to the Hall–Petch strengthening^30^ where a larger number of grain boundaries impede the dislocation movement, which is in agreement with the Ta-Hf-C samples.

**Table 2:** Hardness, elastic recovery, nanowear and corrosion properties (E_corr_ = corrosion potential and I_corr_ = corrosion current) of the films.

Sample	H (GPa)	Elastic recovery (%)	Nanowear volume (μm^3^)	E_corr_ (V)	I_corr_ (µA/cm^2^)	Corrosion rate (μm/year)
TaC	12.3 ± 0.2	69	0.445 ± 0.03	−0.087 ± 0.002	7.750 ± 0.004	206.5 ± 0.1
70TaC-30HfC	24.6 ± 0.3	85	0.199 ± 0.02	−0.19 ± 0.02	0.051 ± 0.002	13.43 ± 0.05
30TaC-70HfC	26.2 ± 0.3	87	0.329 ± 0.03	−0.16 ± 0.01	0.0022 ± 0.0001	0.058 ± 0.01
HfC	18.5 ± 0.5	83	1.19 ± 0.1	−0.020 ± 0.001	131.00 ± 0.01	3481 ± 30

**Figure 5:**
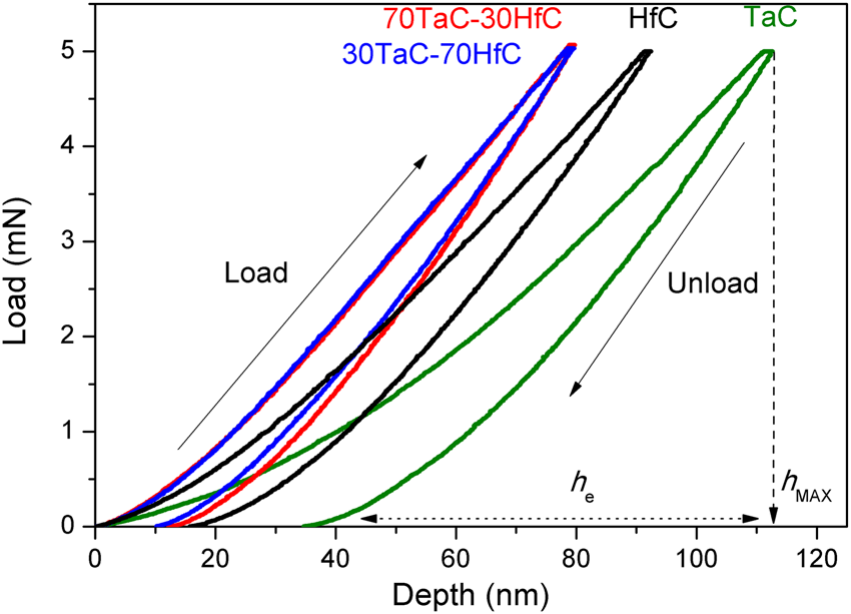
Load-displacement nanoindentation curves of the Ta-Hf-C films, showing the load and unloading regions of the curves. The line-dashed line shows the maximum penetration (*h*_MAX_) of the TaC curve and dot-dashed line shows the elastic recovery region (*h*_e_)

One of the main drawbacks of the TaC and Ta-Hf-C carbides is their brittleness^3, 11^ which restricts their possible applications. The elastic recovery data, extracted from the load-displacement nanoindentation curves, by *h*_e_/*h*_MAX_ shown in Fig. 5, shows that the ternary alloys present a high elastic recovery, reaching a recovery of around 87% for the 30TaC-70HfC sample. The combination of high hardness and elastic recovery in the ternary Ta-Hf-C alloys indicates that the samples are suitable to applications where high loads are required. Additionally, a high elastic recovery is beneficial to compensate for any possible mismatch in the coefficient of thermal expansion between the film and substrate materials, avoiding the formation of cracks and delamination of the film during thermal cycling.

To study the tribological properties of the films we performed nanowear tests. In our study, the nanowear is defined as the total volume removed from the scanned area. The nanowear in the TaC and HfC samples is 0.445 and 1.19 μm^3^, respectively, while in the ternary 70TaC-30HfC and 30TaC-70HfC samples the nanowear decreases to 0.199 and 0.329 μm^3^, respectively. The low nanowear in the ternary alloy films can be associated to their higher hardness and lower nano roughness compared to the binary films.

**Figure 6:**
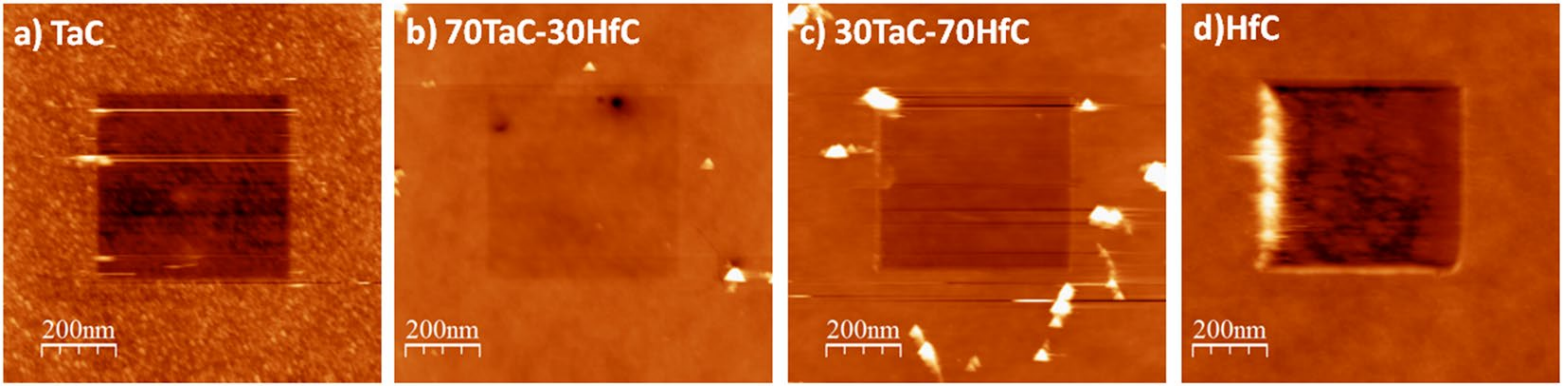
Scanning probe microscopy (SPM) images after nanowear test of the Ta-Hf-C samples. Nanowear tests were performed over an area of 1 µm × 1 µm at a constant load of 0.1 mN and a frequency of 2 Hz (20 µm/s).

In other ternary films made of W-S-C the nanowear tests have been carried out at loads in the order of nN^31^, however, for the Ta-Hf-C alloy films, loads in the order of µN were required to measure detectable wear on all the samples, Fig. 6. This fact, combined with the relatively high hardness and elastic recovery in the ternary alloy films, highlights the outstanding mechanical and nano tribological behavior of the Ta-Hf-C films.

### Electrochemical properties

**Figure 7:**
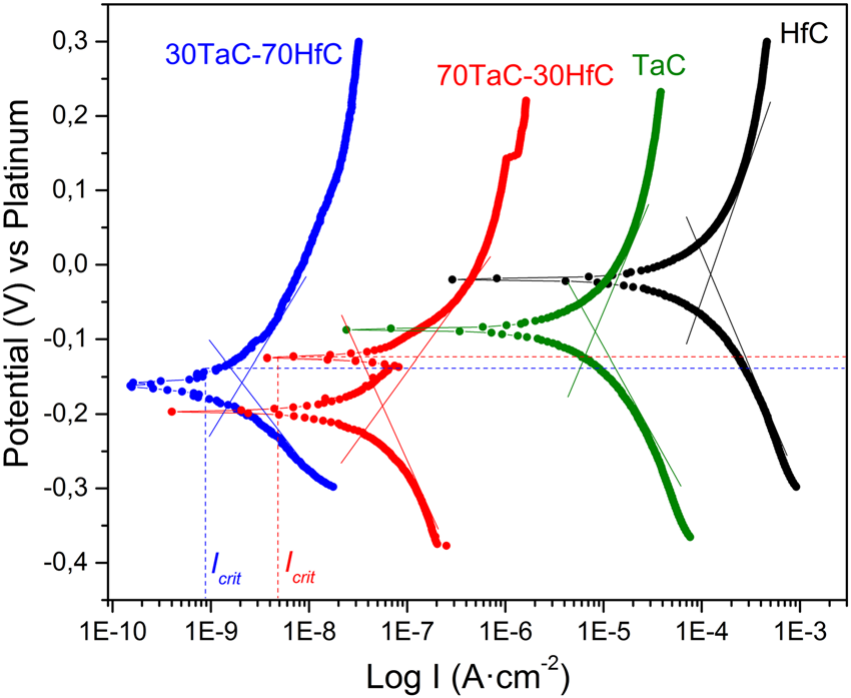
Potentiodynamic polarization curves of the Ta-Hf-C samples at 700 °C. Tafel slopes are drawn on the potentiodynamic curves. The critical current and potentials for 30TaC-70HfC and 70TaC-30HfC samples are also indicated.

Potentiodynamic polarization curves are shown in Fig. 7. The binary and ternary film samples were exposed to the V_2_O_5_-Na_2_SO_4_ molten salt mixture at 700 °C. The more noble potential at 700 °C is attributed to the HfC binary film (where the corrosion potential is shifted to more noble values) compared to the TaC and the ternary alloys. The potentiodynamic curve for the HfC binary film presents a general dissolution in the anodic region, in which there is oxidation and the increasing of the potential does not lead to an increasing in the corrosion current. The TaC binary film shows a potentiodynamic curve with a typical behavior, where the current increases with the potential respect to the stabilization potential, and thus increasing the corrosion rate.

The 30TaC-70HfC ternary alloy exhibits a passivation region after reaching the critical current corresponding to 0.086 μA/cm^2^, with a potential of −0.13 V vs Platinum. This resistance to hot corrosion induces the creation of a passivating layer whose stability is relatively weak, because at −0.10 V vs Platinum, it is observed a gradual increase of the current density. The relevance of this layer is that it can behave as a diffusion barrier for the corrosion reaction products. After reaching a voltage of −0.10 V vs Platinum, it is evidenced a general dissolution.

In the case of the 70TaC-30HfC alloy, we observe that the rate of the dissolution of the film is stabilized after reaching the critical current, 0.00478 μA/cm^2^, where the current density is no longer increasing. In this sample, at a potential of −0.12 V vs Platinum, due to the chemical reactivity of the film in contact with the corrosive salts at 700 °C, there is an increased degradation which may generate a passivation layer. This is observed by the presence of a second current peak suggesting the formation of an oxide layer which protects the alloy film from the hot corrosive environments. Interestingly enough, the experimental set up used in our studies prevents the inclusion of humidity or H^+^ sources, thus relating the second anodic current to the sulfurization/oxidation of 70TaC-30HfC sample^32^. The presence of this passivating layer is in agreement with the observed precipitation of free standing salts, which results both in higher corrosion resistance, when compared with the binary films, and solid lubrication of the surfaces as demonstrated by lubrication studies of P. Gonzalez-Rodriguez *et al*.^33^.

Table 2 shows the hot corrosion properties extracted from the potentiodynamic polarization curves. It is observed that the HfC and TaC binary films offer the lowest corrosion protection among the samples studied. Those samples generate high corrosion current values, which is corroborated in the potentiodynamic polarization curves in Fig. 7, where both TaC and HfC alloys have a larger shift to the right side of the figure, implying an increase in the current dissolution and hence a higher corrosion rate. On the other hand, the ternary Ta-Hf-C alloys presented the lower corrosion rate values, indicating that the ternary alloys are more resistant to corrosion in presence of V_2_O_5_ and Na_2_SO_4_. The sample with the lower corrosion rate, around 0.058 μm/year, was the 30TaC-70HfC ternary alloy. The sample 70TaC-30HfC presents a higher corrosion rate, around 13.43 μm/year. It is possible that a higher diffusion of sulfur at the interface film/oxide, generates the failure of the 70TaC-30HfC film, placing its corrosion resistant properties half way between the TaC film and the 30TaC-70HfC. In general, we assume that the lower corrosion rates in the ternary alloys are attributed to the smaller grain size and less micro/nanopores compared to the binary films that avoid the diffusion of corrosive species from the surface to the interface film/substrate, as suggested by the morphological and the mechanical and tribological tests, where superior hardness and elastic recoveries were found in the ternary alloys.

**Figure 8:**
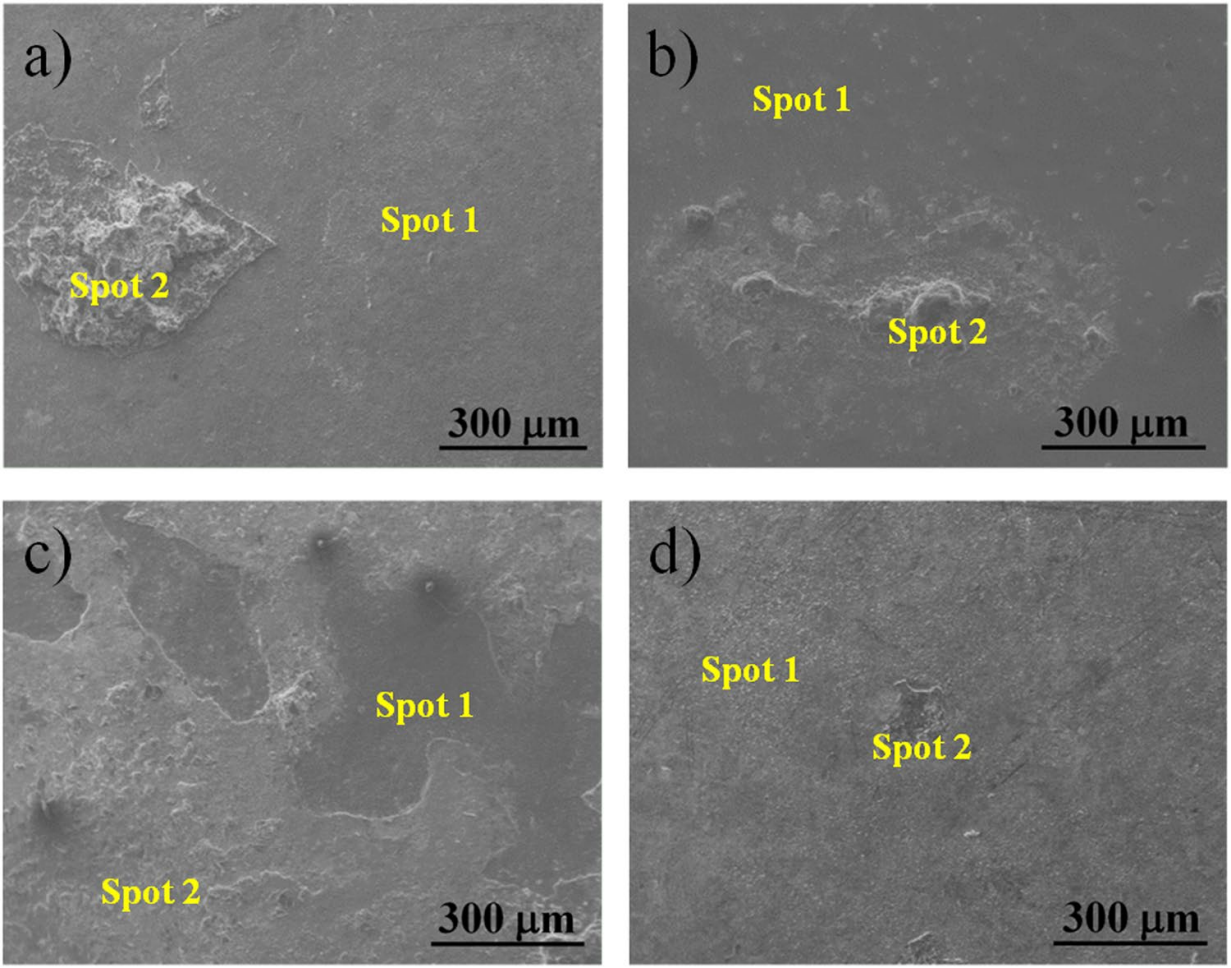
Surface morphology of the Ta-Hf-C samples after exposure to a mixture of pentoxide vanadium, V_2_O_5_, and sodium sulfate, Na_2_SO_4_ at 700 °C; (**a**) TaC, (**b**) 70TaC-30HfC, (**c**) 30TaC-70HfC, and (**d**) HfC.

Figure 8 shows the SEM images of the different Ta-Hf-C samples after being exposed to the mixture of pentoxide vanadium, V_2_O_5_, and sodium sulfate, Na_2_SO_4_ in the corrosion analysis at 700 °C. In general, the film surfaces did not show any cracks or delamination, however, some aggregates of V_2_O_5_ and Na_2_SO_4_, marked in Fig. 8 as spot 2, were dissolved and deposited on the surface of the samples. The formation of these aggregates is in agreement with the low temperature hot corrosion mechanism, where a significant dissolution of some corrosion products is found on the surface of the samples at temperatures between 700–750 °C^17^. The EDS analysis on the aggregates indicated that these are rich in vanadium and sodium, while the surface of the films (measured in spot 1) were rich in carbon, oxygen and sulfur (see Supplementary Table S1). The presence of sulfur on the surface of the films was especially higher in the TaC (Fig. 8a) and HfC (Fig. 8d) films, suggesting that these are more reactive to the Na_2_SO_4_. On the other hand, the ternary 70TaC-30HfC and 30TaC-70HfC alloys (Fig. 8b and c) showed a high amount of oxygen and carbon on the surface of the films (spot 1) suggesting the formation of an oxide-carbon rich coating. The grazing incidence XRD analysis on the Ta-Hf-C samples (see Supplementary Fig. S1) revealed the presence of Ta_2_O_5_ (JCPDS-ICDD 04-007-0607) and HfO_2_ (JCPDS-ICDD 04-014-7409) oxides, together with a minor amount of the TaC and HfC phases. This clearly demonstrates that the thin films were not dissolved and shows the formation of passive oxide surfaces protecting the steel substrates from the corrosion.

SEM and XRD analysis show that the corrosion is generated by the V_2_O_5_ and Na_2_SO_4_ salts, and consequently these salts are deposited on the surface of the samples during the corrosion tests thus generating an adequate performance and avoiding accelerated corrosion due to pitting corrosion processes normally observed in pure metals. The Ta-Hf-C alloys form passive surfaces and increase the protection of the system by creating a layer and preventing the formation of volatile oxides from the metal.

Finally, and in order to apply the Ta-Hf-C coatings as thermal protective coatings or thermal barrier coatings (TBC), these materials have to be deposited on other metallic layers acting as bond coats between the TBC and the substrate. The most common bond coats are those based on metallic MCrAlY alloys (where M=Co, Ni or Ni-Co mixtures)^34^ and the basic rule to avoid cracks or delamination between the thermal protective coating on top and the bond coat is the matching of the coefficients of thermal expansion (CTE) of both materials. It has been recently found that the CTE of binary and ternary alloys for the Ta-Hf-C system varies from 7.08 to 7.66·10^−6^/K in the range from 25 to 2000 °C^35^, which match the CTE values for some common MCrAlY alloys in the range of 500 to 700 °C^34^, which is a similar temperature range as the used in this work to study the corrosion behavior of the Ta-Hf-C samples. In addition, the high thermal conductivities reported for TaC^36^ and Ta-Hf-C^35^ carbides ensure an enhanced heat dissipation for applications that requires working at high temperatures such as turbine blades or hypersonic flights^35^.

## Conclusions

In this study we have successfully obtained binary and ternary thin films of the Ta-Hf-C system with outstanding tribo-mechanical and corrosion resistance properties. We have also investigated and reported for the first time their hot anti-corrosion behavior in V_2_O_5_-Na_2_SO_4_ salts at 700 °C. We found that the ternary alloys consist of a solid solution of the binary TaC and HfC carbides (Ta-Hf-C), as shown by the XRD and XPS analysis. The ternary alloys showed superior hardness and elastic recovery, with values greater than 24.6 GPa and 85%, respectively, an important improvement when compared with results found for the binary carbide films.

Finally, the corrosion rates extracted from the potentiodynamic curves showed that the ternary alloys have a superior anticorrosion behavior compared to the binary films, with corrosion rates as low as 0.058 μm/year.

The combination of high hardness and elastic recovery, low nanowear and an excellent resistance to hot corrosion demonstrates the potential of the ternary Ta-Hf-C alloy films for protecting applications where extreme conditions are demanded such as turbine blades and steam generators.

## Electronic supplementary material

Supplementary Information
