# Supplementary material for: Robust tribo-mechanical and hot corrosion resistance of ultra-refractory Ta-Hf-C ternary alloy films
Source: Sci Rep. 2017 Jun 8;7:3080. doi: 10.1038/s41598-017-03181-2 (PMC5465092; doi:10.1038/s41598-017-03181-2)
Supplement: Supplementary file 1 — Supplementary Information [file 41598_2017_3181_MOESM1_ESM.pdf]

Supplementary Information

Robust tribo-mechanical and hot corrosion resistance of ultra-refractory Ta-Hf-C ternary alloy films

Luis Yate[1]*, L. Emerson Coy[2]**, Willian Aperador[3]

[1] *CIC biomaGUNE, Paseo Miramón 182, 20009 Donostia-San Sebastian, Spain*

[2] *NanoBioMedical Center, Adam Mickiewicz University, Umultowska 85, 61-614 Poznan, Poland*

[3] *School of Engineering, Universidad Militar Nueva Granada, Carrera 11 #101-80, 49300 Bogotá, Colombia*

* Corresponding Author. Tel: +34 943 00 53 37. E-mail: lyate@cicbiomagune.es

** Co-Corresponding Author. Tel +48 61 829 6704. E-mail: coyeme@amu.edu.pl

Table S1. Relative chemical composition of the samples (obtained by EDS) after exposure to a mixture of pentoxide vanadium, V_2O_5, and sodium sulfate, Na_2SO_4 at 700ºC. Measurements on spot 1 are made on the surface of the film, while measurements on spot 2 are made on the aggregates.

Element (at.%)	TaC		70TaC-30HfC		30TaC-70HfC		HfC	
	Spot 1 (film)	Spot 2 (aggregate)	Spot 1 (film)	Spot 2 (aggregate)	Spot 1 (film)	Spot 2 (aggregate)	Spot 1 (film)	Spot 2 (aggregate)
**C**	31	18	31	25	77	8	37	7
**O**	47	55	67	52	20	65	44	69
**Na**	4	9	-	11	-	11	-	5
**S**	18	5	-	11	3	9	19	9
**V**	-	12	2	2	-	7	-	10

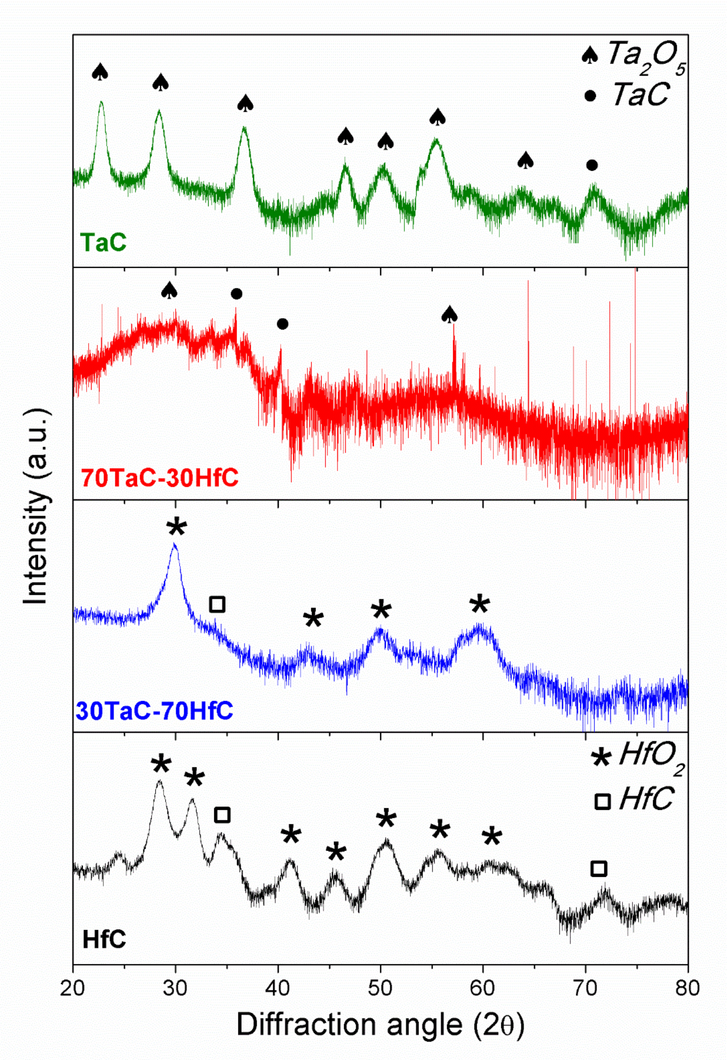

Figure S1. Grazing incidence XRD patterns of the Ta-Hf-C samples after being exposed to the mixture of pentoxide vanadium, V_2O_5, and sodium sulfate, Na_2SO_4 in the corrosion analysis at 700ºC.
